# The role of physical activity in preventing cognitive decline among U.S. older adults with diabetes and prediabetes: a cross-sectional study

**DOI:** 10.3389/fpubh.2025.1603627

**Published:** 2025-06-24

**Authors:** Yifei Wang, Dezheng Liu, Hongli Wang, Mengzhao Wang, Weiqi Ruan, Yanbai Han, Yiming Han

**Affiliations:** ^1^College of Physical Education and Health, Guangxi Normal University, Guilin, China; ^2^Faculty of Education and Liberal Studies, City University, Petaling Jaya, Malaysia; ^3^Faculty of Kinesiology, Sport, and Recreation, University of Alberta, Edmonton, AB, Canada

**Keywords:** physical activity, cognitive decline, older adults, diabetes, prediabetes, NHANES

## Abstract

**Background:**

Physical activity (PA) has been widely recognized as a key strategy to slow age-related cognitive decline. However, its specific effects on older adults with diabetes or prediabetes remain poorly understood. Therefore, we investigated the association between different levels of PA and cognitive function among older Americans with diabetes and prediabetes.

**Methods:**

This cross-sectional study used data from the 2011–2014 National Health and Nutrition Examination Survey (NHANES) and included a total of 1,299 older adults aged ≥60 years. The PA levels were determined by calculating the weekly metabolic equivalent of task time (MET-min/week). The participants’ cognitive abilities were assessed using the Consortium to Establish a Registry for Alzheimer’s disease (CERAD) Word Learning Test, Animal Fluency Test (AFT), and Digit Symbol Substitution Test (DSST). Multivariable logistic regression models were used to analyze the association between different PA levels and cognitive function in patients with diabetes and prediabetes. The study utilized the restricted cubic spline (RCS) models to explore the nonlinear correlation of PA with cognitive function.

**Results:**

Upon controlling for confounders, DSST scores were still significantly associated with moderate-level PA (OR: 0.457, 95% CI: 0.244, 0.853, *p* = 0.020) and high-level PA (OR: 0.478, 95% CI: 0.240, 0.955, *p* = 0.039). According to the RCS models, PA showed a significant nonlinear correlation with cognitive function, and the risk of cognitive decline decreased with the increase of PA levels.

**Conclusion:**

In older adults with diabetes and prediabetes, moderate and high levels of physical activity are associated with a lower risk of cognitive decline. Clinicians should encourage patients to participate actively in exercise to maximize the benefits of PA.

## 1 Introduction

Dementia, a syndrome caused by a variety of diseases, would wreak havoc on nerve cells and damage the brain over time, leading to deterioration in cognitive function. With a great impact, dementia is one of the main causes of the older adult’s incapacity and dependence on others (1). Currently, there are over 55 million dementia cases globally, with estimates suggesting a threefold rise to more than 150 million by 2050 (2). The United States is one of the countries in the world with the highest burden of diabetes and prediabetes among the older adult (3). The prevalence rate is increasing year by year, and approximately 10% of patients with prediabetes progress to diabetes each year (4). Cognitive decline is an important precursor to the onset of dementia. Numerous studies have shown that both diabetes and prediabetes contribute to a greater susceptibility to cognitive decline and dementia (5, 6). Various neuropathological mechanisms can explain this association, such as large production of *β*-amyloid and impaired cerebral blood flow caused by disruption of cellular metabolism due to insulin resistance or inadequate secretion (7). In addition, in studies of older adults, it was found that patients in the diabetic or pre-diabetic stage had reduced brain volume and elevated HbA1c, and this led to significant declines in cognitive domains such as memory, attention and executive function, which in turn increased risk of cognitive decline and thus dementia (8–10). Among the many dementia assessment tools, the Consortium to Establish a Registry for Alzheimer’s Disease (CERAD) Word Learning Test, Animal Fluency Test (AFT), and Digit Symbol Substitution Test (DSST) have been widely used, not only for diagnosing dementia but also for intuitively evaluating overall cognitive function (11).

Physical activity (PA) is defined as any motion that involves the bones and muscles, and such physical action will result in energy consumption. PA may also enhance physical fitness and promote physical and mental health (12). In the general population, PA is an effective means of preventing cognitive decline and reducing the risk of developing dementia (13, 14). Research indicates that engaging in more PA can lower the chances of developing dementia and offset the harmful consequences of a sedentary lifestyle (15). Another study has shown that, compared to inactive individuals, more physical activity among older adults reduced the risk of cognitive impairment and made them perform better on tests of overall cognitive and executive function (16). Furthermore, PA may confer multiple benefits to patients with diabetes or prediabetes, including improved insulin sensitivity, improved glycemic control, and improved cardiovascular health (17–19). At present, some studies have explored the impact of PA on cognitive function among individuals with diabetes, suggesting that a physically active lifestyle is associated with a slower rate of cognitive decline and that aerobic exercise can improve overall cognitive performance in older adults with diabetes (20, 21). However, to date, no study has utilized a nationally representative U.S. sample to examine the association between PA and multidomain cognitive function among older adults with diabetes or prediabetes. Furthermore, the dose–response relationship between PA and cognitive performance remains unclear. Therefore, investigating the effects of varying levels of PA on cognitive function in older adults with diabetes or prediabetes not only helps to elucidate the role of PA in maintaining or improving cognitive health, but also contributes to enhancing their quality of life and potentially preventing the onset of dementia.

The objective of this study is to investigate the association of PA with cognitive function among older adults with diabetes and prediabetes by employing a sample of a nationally representative U.S. population, thereby providing important guidelines for clinicians and patients.

## 2 Materials and methods

### 2.1 Study population

Data for this study were obtained from the National Health and Nutrition Examination Survey (NHANES), a biennial representative sample survey of the U.S. population conducted by the Centers for Disease Control and Prevention since 1999 using a cross-sectional, stratified, multi-stage probability sampling design to assess the health and nutritional status of the U.S. population (22). All NHANES survey protocols have been approved by the Ethics Review Board of the National Center for Health Statistics and all of the participants offered written informed consent (23).

**Figure 1:**
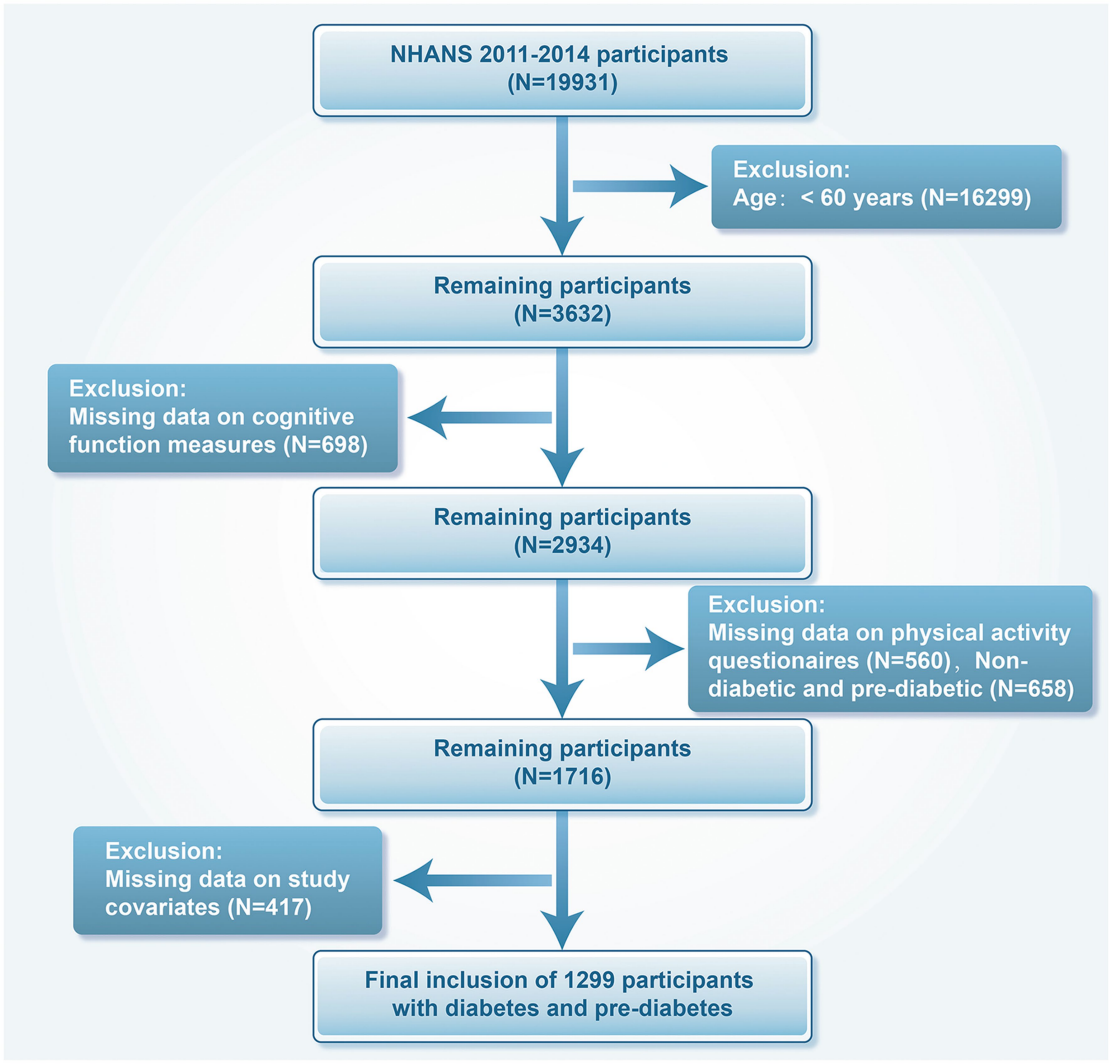
Flowchart for screening research participants.

We included data from the NHANES cycles of 2011–2012 and 2013–2014, comprising 19,931 individuals aged 60 years and above with prediabetes or diabetes. We excluded 16,299 patients younger than 60 years of age, 698 with missing cognitive function measures, 560 with missing PA questionnaires, 658 patients without diabetes and prediabetes, and 417 with missing covariates. In the end, 1,299 participants were analyzed (Figure 1).

### 2.2 Diabetes and prediabetes

Diabetic status is self-reported as diagnosed by a physician or healthcare practitioner, or as determined by higher levels of fasting blood glucose (FBG) (≥ 126 mg/dL), blood glucose 2 h post oral glucose tolerance test (≥ 200 mg/dL) and HbA1c (≥ 6.5%). Prediabetes identifies who does not have diabetes but meets one or more of the following criteria: prediabetes self-reported as diagnosed by a physician or healthcare practitioner, a FBG concentration of 100–125 mg/dL, blood glucose of 140–199 mg/dL 2 h post oral glucose tolerance test, or HbA1c at 5.7–6.4% (24).

### 2.3 MET and PA calculation

Metabolic equivalent of task (MET) is a measure of the oxygen needed to sustain basic metabolism and is frequently used to gage relative energy metabolism and exercise intensity in various activities (25). In this study, the Global Physical Activity Questionnaire (GPAQ) was used to evaluate PA via MET to represent exercise intensity (26). The PAQ survey involved the following PA: vigorous work-related (MET = 8.0), vigorous leisure-time (MET = 8.0), moderate work-related (MET = 4.0), walking or bicycling for transportation (MET = 4.0), and moderate leisure-time (MET = 4.0). By multiplying MET, activity type, weekly frequency, and duration of each session, MET-min/week was calculated, and the PA levels of each participant were quantified accordingly (27). PA levels (MET-min/week) were categorized into four groups according to the Physical Activity Guidelines for Americans (28): (1) no PA (No-PA): 0, (2) low-level PA (LLPA): 1–599, (3) moderate-level PA (MLPA): 600–1,200, and (4) high-level PA (HLPA): >1,200.

### 2.4 Cognitive function

Participants (60 years of age and above) in the NHANES survey conducted from 2011 to 2014 underwent three cognitive tests. The Immediate Word Learning (CERAD-WL) and Delayed Recall Module (CERAD-DR), the AFT, and the DSST of the CERAD were established.

The CERAD Word Learning subtest assessed the immediate and delayed ability to learn new language information (29). The test consists of three sequential learning trials and one delayed recall. Participants were tasked with reading 10 unrelated words out loud in the learning trials, one word at a time. Following the appearance of the words, the participant promptly recollected as many words as they could. The sequence of the 10 words was altered in each of the three learning trials. After finishing the AFT and DSST tasks, a delayed word recall test was conducted. The CERAD test comprised three learning trials and one delayed recall test, with each test scored on a scale of 0–10, and the total score being the sum of all four tests. The AFT assessed the ability to fluently categorize speech, which was a key aspect of executive function. Participants were asked to list as many animals as possible in 1 min, earning one point for each animal named (30). The DSST was a component of the Wechsler Adult Intelligence Scale (WAIS III) that assessed processing speed, sustained attention, and working memory (31). In each dimension of cognitive function, lower scores indicate poorer cognitive function.

Based on the previous study, the ages of 60–69, 70–79, and ≥80 were, respectively, set as three age groups to correspond to the lower cut-off values for different cognitive tests (32). The cut-off values for low cognitive performance in the three age groups were as follows: 22, 19, and 16 for the CERAD test; 14, 13, and 12 for the AFT; and 38, 34, and 29 for the DSST.

### 2.5 Covariates assessment

Based on prior studies, the following covariates were included to reduce the effect of potential confounders (33–36). Demographic characteristics, including age (60–69, 70–79, 80 and older), gender (male/female), race (Mexican-American, non-Hispanic white, non-Hispanic black, and other races), education (< high school diploma, high school graduate/equivalent, >high school diploma), marital status (Married, Widow/divorce/separation, Unmarried and Cohabitation) and household poverty-to-income ratio (PIR), were obtained from the NHANES survey. Smoking status was classified as never smokers, former smokers and current smokers; a participant was considered a drinker if he or she had consumed alcohol more than 12 times in 1 year. The body mass index (BMI) was determined by professional technician in physical examination by dividing the weight in kilograms by the square of the height in meters. Diet quality was assessed using the Healthy Eating Index-2015 (HEI) score, and higher scores indicated healthier diet. The nine-item Patient Health Questionnaire (PHQ-9) was used to evaluate depression (score ≥10) (37). Hypertension, stroke, and coronary heart disease (CHD) were confirmed by physician or health care professional.

### 2.6 Statistical analysis

Sample weights provided by NHANES were used to weight all statistical analyses in this study, and the stratification and clustering methods for complex sampling designs were employed. The analysis strictly adhered to NHANES guidelines. A weighted presentation was used to illustrate basic characteristics traits of the study population for a nationally representative estimate (38). Continuous variables that were not normally distributed were reported as median interquartile range, while categorical variables were shown as N (%). Multivariate logistic regression analysis was performed to analyze the associations between different levels of PA and three cognitive function tests in patients with diabetes or prediabetes, and the results were presented as odds ratios (OR) with 95% confidence intervals (CI). Model 1 did not adjust for confounding factors; gender, age, ethnicity, education, marital status and PIR were added to Model 2; and Model 3 further adjusted for HEI, BMI, smoke, drink, hypertension, CHD, stroke, and depression on the basis of Model 2. Secondly, we employed the restricted cubic spline (RCS) model to analyze the nonlinear relationship between the continuous variable PA and cognitive function. The number of nodes was set at 3, and the goodness of fit of the model was verified using the Akaike Information Criterion (AIC). Finally, subgroup analyses and interaction tests were conducted to investigate the association of PA with cognitive function across various populations. Based on the criteria of whether meeting the recommended amount of PA guidelines (39), the participants were assigned to two groups and classified by age, sex, race/ethnicity, marital status, education, smoking, drinking, hypertension, depression, diabetes, and prediabetes.

The R software (4.3.0 version) was utilized for the statistical analysis. All statistical tests were two-sided, and statistical significance was determined at a threshold of *p* < 0.05.

## 3 Results

### 3.1 Baseline characteristics of participants by PA level

A total of 1,299 older adults with diabetes or prediabetes (weighted population 22,959,343) were included in the final analysis. In the study population, the proportion of patients aged 60 to 69 was 56%; that of patients aged 70 to 79 was 29%, and that of patients aged over or equal to 80 was 15%. In terms of gender, 48% were male, and 52% were female. In regards to race, the proportion of Mexican American, Non-Hispanic White, Non-Hispanic Black, and Others was 3.7, 78, 9.1, and 9.2%, respectively. About 61% of the participants were married, and 58% had a high school degree or higher. Drinkers and current smokers accounted for 73 and 10%, respectively. Table 1 illustrates the baseline traits of the weighted samples.

**Table 1:** Baseline characteristics of participants by physical activity level, from NHANES (2011–2014).

Characteristic	*N*^1^	Overall, *N* = 22,959,343^2^	No physical activity, *N* = 10,223,341^2^	Low-level physical activity, *N* = 3,184,643^2^	Moderate-level physical activity, *N* = 3,182,347^2^	High-level physical activity, *N* = 6,369,012^2^	*p*-value^3^
Gender	1,299						0.007
Male		637 (48%)	256 (43%)	101 (42%)	84 (45%)	196 (60%)	
Female		662 (52%)	334 (57%)	115 (58%)	85 (55%)	128 (40%)	
Age	1,299						<0.001
60–69		707 (56%)	287 (50%)	119 (52%)	94 (55%)	207 (67%)	
70–79		379 (29%)	188 (31%)	65 (31%)	40 (26%)	86 (25%)	
≥80		213 (15%)	115 (19%)	32 (17%)	35 (18%)	31 (7.4%)	
Race	1,299						0.003
Mexican American		120 (3.7%)	56 (3.9%)	20 (4.4%)	14 (3.2%)	30 (3.3%)	
Non-Hispanic White		603 (78%)	287 (78%)	81 (67%)	86 (82%)	149 (81%)	
Non-Hispanic Black		321 (9.1%)	153 (9.9%)	63 (14%)	35 (6.1%)	70 (6.9%)	
Other Race		255 (9.2%)	94 (8.2%)	52 (14.6%)	34 (8.7%)	75 (8.8%)	
Education	1,299						0.013
<High school diploma		339 (19%)	179 (23%)	50 (16%)	44 (20%)	66 (13%)	
High school diploma/equivalent		314 (23%)	160 (26%)	57 (25%)	32 (21%)	65 (18%)	
>High school diploma		646 (58%)	251 (51%)	109 (59%)	93 (58%)	193 (69%)	
Marital	1,299						0.344
Married		722 (61%)	296 (56%)	120 (61%)	104 (68%)	202 (66%)	
Widow/divorce/separation		461 (31%)	240 (35%)	79 (33%)	54 (24%)	88 (26%)	
Unmarried		85 (5.4%)	42 (5.9%)	11 (3.8%)	9 (6.0%)	23 (5.0%)	
Cohabitation		31 (3.0%)	12 (3.8%)	6 (2.4%)	2 (1.4%)	11 (2.9%)	
Smoke	1,299						0.311
Never		626 (48%)	280 (47%)	101 (49%)	89 (51%)	156 (48%)	
Former		521 (42%)	229 (40%)	93 (44%)	62 (40%)	137 (45%)	
Current		152 (10%)	81 (14%)	22 (7.6%)	18 (9.3%)	31 (7.1%)	
Drink	1,299	886 (73%)	384 (68%)	154 (79%)	116 (70%)	232 (79%)	0.037
PIR	1,299	2.75 (1.60, 4.76)	2.31 (1.30, 4.25)	2.64 (1.53, 4.62)	2.84 (1.74, 4.49)	3.65 (2.08, 5.00)	<0.001
BMI	1,299	29 (26, 34)	30 (26, 35)	29 (25, 33)	28 (26, 32)	28 (25, 31)	<0.001
HEI	1,299	54 (46, 62)	52 (45, 59)	56 (47, 64)	56 (46, 63)	57 (48, 64)	0.003
Hypertension	1,299	863 (64%)	427 (69%)	138 (62%)	110 (63%)	188 (57%)	0.079
CHD	1,299	143 (12%)	72 (14%)	22 (7.8%)	27 (16%)	22 (8.3%)	0.087
Stroke	1,299	96 (7.0%)	59 (10%)	11 (4.7%)	11 (4.8%)	15 (4.3%)	0.061
Depression	1,299	135 (8.7%)	75 (12%)	27 (10%)	11 (4.7%)	22 (5.4%)	0.027
CERAD	1,299	26 (22, 30)	25 (21, 30)	25 (21, 30)	28 (23, 32)	26 (22, 30)	0.017
AFT	1,299	18 (14, 21)	16 (13, 20)	17 (14, 20)	19 (15, 23)	19 (15, 23)	<0.001
DSST	1,299	50 (40, 63)	46 (35, 58)	50 (39, 61)	53 (44, 67)	56 (46, 66)	<0.001

^1^ N not Missing (unweighted).

^2^ N (unweighted) (%); Median (25%, 75%).

^3 chi-squared test with Rao & Scott’s second-order correction; Wilcoxon rank-sum test for complex survey samples.^

Superscript 2: weighted sample size.

PIR, poverty income ratio; BMI, body mass index; HEI, healthy eating index; CHD, coronary heart disease; CERAD, Consortium to Establish a Registry for Alzheimer’s Disease; AFT, Animal Fluency Test; DSST, Digit Symbol Substitution Test; NHANES, National Health and Nutrition Examination Survey.

### 3.2 Association between physical activity level and cognitive function

Associations were found between various levels of PA and three cognitive function tests based on the weighted logistic regression model. In Model 1, there was a significant association between HLPA and CERAD scores compared with the No-PA group (OR: 0.621, 95% CI: 0.388, 0.994, *p* = 0.047). After adjusting for other confounding factors, the protective effect of PA on cognitive function was not observed in Model 2 and Model 3. In the AFT, participants who reported LLPA (OR: 0.571, 95% CI: 0.364, 0.894, *p* = 0.016), MLPA (OR: 0.365, 95% CI: 0.172, 0.775, *p* = 0.011), and HLPA (OR: 0.519, 95% CI: 0.284, 0.949, *p* = 0.034) were significantly associated with outcomes in Model 1 compared with the No-PA group; after adjusting for Model 2, LLPA (OR:0.513, 95% CI: 0.310, 0.850, *p* = 0.032) and MLPA (OR:0.400, 95% CI: 0.179, 0.894, *p* = 0.028) were still significantly correlated with cognitive ability (*p* < 0.05); after adjusting for Model 3, no significant association between PA and AFT scores was observed. In the DSST, participants with MLPA and HLPA were significantly associated with higher cognitive function scores in Model 1, Model 2, and Model 3 compared to the No-PA group (*p* < 0.05). In the fully adjusted model, the risk of cognitive decline in the moderate PA group and the high PA group was reduced by 54.3 and 52.2%, respectively (Table 2).

**Table 2:** Associations between different levels of physical activity and three measures of cognitive function.

Cognitive function tests	Model 1			Model 2			Model 3		
	OR	95% CI	*p*-value	OR	95% CI	*p*-value	OR	95% CI	*p*-value
CERAD									
No-PA	Reference			Reference			Reference		
Low-level PA	0.956	0.516, 1.769	0.881	1.092	0.616, 1.935	0.750	1.142	0.647, 2.019	0.610
Moderate-level PA	0.602	0.348, 1.042	0.068	0.702	0.407, 1.212	0.190	0.697	0.387, 1.254	0.197
High-level-PA	0.621	0.388, 0.994	0.047	0.807	0.501, 1.301	0.358	0.791	0.476, 1.314	0.323
AFT									
No-PA	Reference			Reference			Reference		
Low-level-PA	0.571	0.364, 0.894	0.016	0.513	0.310, 0.850	0.032	0.566	0.333, 1.061	0.068
Moderate-level-PA	0.365	0.172, 0.775	0.011	0.400	0.179, 0.894	0.028	0.442	0.186, 1.053	0.062
High-level-PA	0.519	0.284, 0.949	0.034	0.670	0.361, 1.245	0.191	0.777	0.401, 1.508	0.412
DSST									
No-PA	Reference			Reference			Reference		
Low-level-PA	0.738	0.505, 1.080	0.114	0.739	0.507, 1.079	0.110	0.784	0.503, 1.222	0.246
Moderate-level-PA	0.374	0.211, 0.664	0.002	0.422	0.232, 0.769	0.007	0.457	0.244, 0.853	0.020
High-level-PA	0.319	0.178, 0.571	<0.001	0.438	0.226, 0.850	0.017	0.478	0.240, 0.955	0.039

Model 1: unadjusted.

Model 2: adjusted for age, gender, race/ethnicity, education, marital, PIR.

Model 3: adjusted for Model 2 + HEI, BMI, smoke, drink, hypertension, CHD, stroke, depression.

BMI, body mass index; CHD, coronary heart disease; HEI, healthy eating index; PIR, poverty income ratio; PA, physical activity.

**Figure 2:**
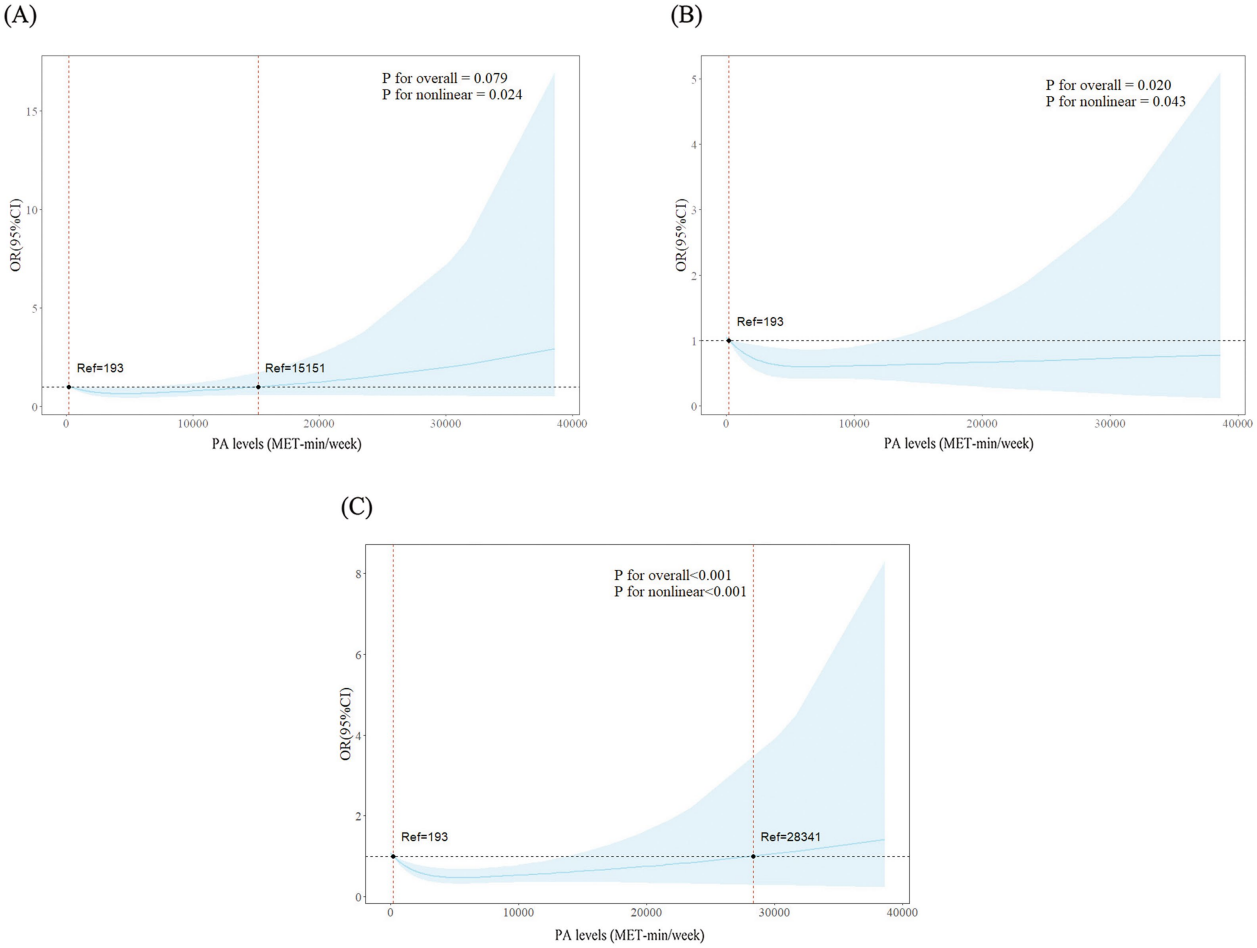
Dose–response associations between physical activity levels and three tests of cognitive function. **(A)** Consortium to establish a registry for Alzheimer’s disease test. **(B)** Animal fluency test. **(C)** Digit symbol substitution test.

The RCS results showed significant nonlinear dose–response correlations between PA levels and CERAD test (*p* for overall = 0.079, *p* for nonlinear = 0.024), AFT (*p* for overall = 0.020, *p* for nonlinear = 0.043), and DSST (*p* for overall<0.001, *p* for nonlinear <0.001). According to the results of CERAD, AFT and DSST, cognitive function benefited the most when the PA reached 6,200 MET-min/week, 4,600 MET-min/week and 5,400 MET-min/week (Figure 2).

### 3.3 Subgroup analyses

**Figure 3:**
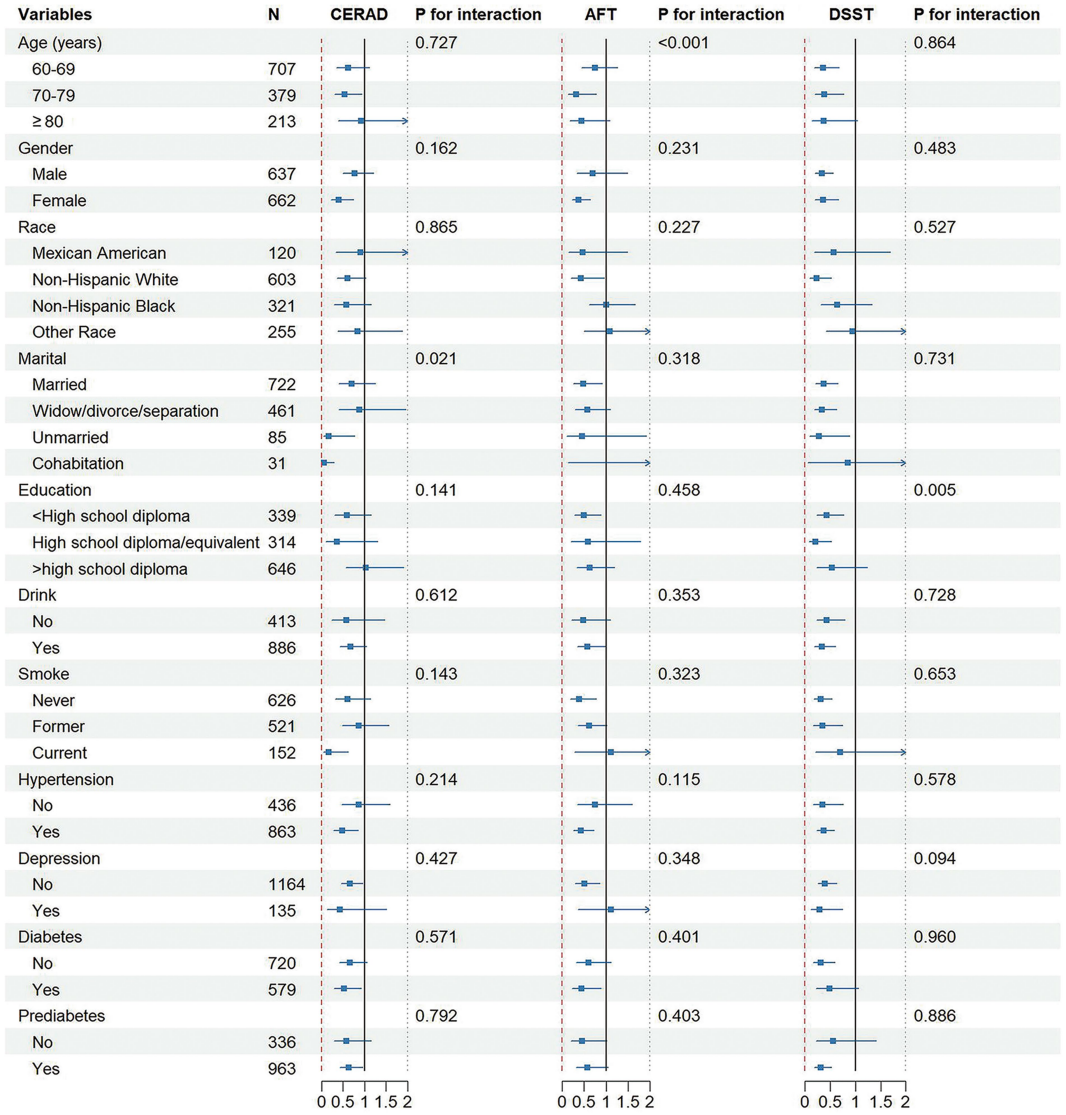
Associations between physical activity levels and cognitive function in different subgroups.

Figure 3 illustrates the associations between PA and cognitive function in different subgroups. The association of PA with cognitive function stayed consistent across various subgroups of variables, such as age, sex, race/ethnicity, education, drink, smoke, hypertension, depression, and diabetes/prediabetes, when using CERAD as the outcome; on the contrary, interaction analysis showed that marital status could affect the association of PA with cognitive ability (*p* interaction = 0.021). With AFT as the outcome indicator, the association between PA and cognitive level remained consistent in subgroups such as gender, ethnicity, marital marriage, educational attainment, drink, smoke, hypertension, depression, diabetes, and prediabetes; in the subgroup of age, the interaction between PA and cognitive level was significant (*p* interaction < 0.001). With DSST as the outcome indicator, educational level was found to influence the correlation of PA with cognitive function (*p* interaction = 0.005) and it remained stable in other subgroups.

## 4 Discussion

In this study of older adults with diabetes and prediabetes in America, it was found that moderate to high levels of PA were associated with better cognitive performance and a lower prevalence of cognitive impairment. After controlling for sociodemographic factors, lifestyle behaviors, and prevalent chronic conditions, a significant correlation between MLPA and HLPA groups and DSST scores was still observed. DSST is recognized as a reliable measure of overall cognitive function. According to our study, maintaining a moderate to high level of physical activity on a weekly basis is associated with a lower risk of cognitive decline and PA could be used as a reliable measure for patients with diabetes or prediabetes to maintain cognitive function.

In America, about 53% of adults do not meet the exercise levels recommended in PA guidelines (40). Engaging in routine PA can offer many benefits to the structure and function levels of brain, including slowing down brain aging, increasing brain volume, and providing potential neuroprotection, which is extremely effective in the older adults (41, 42). PA can improve cognitive levels in individuals with disorders that impair cognitive function, such as schizophrenia, Parkinson’s disease, and stroke (43). We have also found such benefits in patients with diabetes or prediabetes. Previous studies have shown that PA can be used as a long-term protective factor against dementia and cognitive decline in the general population (14); and another randomized controlled trial also found this association, i.e., moderate PA could significantly improve verbal memory, executive function, and overall cognition in healthy older adults (44). Furthermore, the cognitive function of the senior was also shown to be associated with the type of PA, where closed exercise was associated with better selective attention and visuospatial function and open exercise was associated with better inhibitory control and cognitive flexibility (45). These provided a possible explanation for our study.

Currently, few studies have highlighted the importance of PA in preventing cognitive impairment in diabetic patients. A large cohort study in Sweden showed that an active lifestyle (including physical exercise and social activities) may reduce the harmful effects of diabetes on the brain, retain brain volume and reduce the risk of diabetes-related dementia by 30% (46). Another cross-sectional study of senior Chinese patients with type 2 diabetes showed that PA could delay the onset of cognitive impairment possibly due to improved sleep quality and reduced depressive symptoms (47). According to another multicenter cohort study conducted in Brazil, for the middle-aged and older patients with diabetes, leisure time PA was a key moderating factor in cognitive decline that weakened the harmful association between diabetes and cognitive impairment (35). This is the first time we have found this association in seniors in America. Our findings stressed the importance of public health policies that promote the participation of populations at high risk of cognitive impairment and dementia (including patients with diabetes and prediabetes) in PA.

The CERAD and AFT are highly dependent on language ability and vocabulary reserve; however, their results lost statistical significance after adjusting for confounding factors, which may be attributed to the strong influence of educational level and depressive symptoms on language-related cognitive functions (48, 49). In contrast, the DSST primarily reflects processing speed and attention, which are more directly influenced by the neurovascular and metabolic regulation of PA, such as increased cerebral blood flow and the release of brain-derived neurotrophic factor (43). These findings suggest that different cognitive domains may exhibit domain-specific responsiveness to PA. Future studies should employ path analysis to further elucidate the underlying mechanisms.

In the RCS curves, we found that PA levels showed a nonlinear correlation with cognitive function in diabetes and prediabetes patients. According to the results of three cognitive function tests, older patients benefited from low-dose PA, and the risk of their cognitive impairment decreased as PA levels increased. Previous studies have also supported our view that when the overall PA level reaches 3,000–4,000 MET/min/week, it can significantly reduce the risk of various diseases such as diabetes, heart disease, and stroke (50). In terms of preventing cognitive decline, high levels of PA are better than low to medium levels (13); a study by Endeshaw et al. came to a similar conclusion that participation in PA had a positive effect on cognitive function in older adults, and the effects of medium to high levels of PA are better than that of low levels of PA (51). However, the overall activity level of old patients should not be too high. As can be seen from the results of the CERAD test, PA no longer has a protective effect on cognitive function when the PA level is above 15,151 MET-min/week. At extremely high PA levels, the benefits will gradually stabilize and begin to reverse, which may reflect several biological phenomena. First of all, excessive PA may cause fluctuations in the patient’s blood glucose levels, leading to an increase in oxidative stress and inflammatory responses within the brain (52). These inflammatory cytokines can cross the blood–brain barrier, exacerbate neuroinflammation (53). Furthermore, overexercise without sufficient recovery can downregulate brain-derived neurotrophic factor signaling, blunting the neuroplastic adaptations that underlie exercise’s protective effects, and thereby negatively affect cognitive function (54).

In addition, variables (such as age, marital status, and education) were found to play a role in affecting the correlation of PA with cognitive function in our subgroup analysis. The brain of older adults will experience some degenerative changes with age, such as synaptic degeneration, neuronal apoptosis, reduced brain volume, and suffer from cognitive decline. Meanwhile, the nervous system of the older adult will also change; for example, nerve fibers become more fragile and prone to fracture, and the number of neurons will decrease, which will affect the normal function of cognitive function (55, 56). A significant association between cognitive impairment and marital status was also noted. A longitudinal study on the older adult in America suggested that divorce or widowhood may be a risk factor for cognitive impairment and its progression to dementia (57). Furthermore, abundant evidence showed that education also affects the level of cognitive function. In terms of cognitive performance, individuals with a high level of education have a significant advantage over those with a low level of education (48) and the risk of dementia decreases as the level of education increases (58). It was noted in our study that PA had a significant protective effect in <high school diploma group and High school diploma group, and the study by Li et al. also supported our view that PA may contribute to a significant improvement in overall cognition of people with low education level (59). This correlation is no longer significant among the highly educated group. This might be because higher education levels enhance the tolerance to the PA effect through cognitive reserves (48). Future research could combine cognitive training with PA to increase the cognitive benefits for the highly educated population. Moreover, we also found significant benefits of PA in patients with hypertension and depression, possibly because PA helped regulate blood pressure, improve mood and alleviate depressive symptoms, improve memory and concentration of patients, and thus reduce the impairment of cognitive function (49, 60).

It has been inferred that there may be multiple underlying mechanisms for the improvement of cognitive function in older adults with diabetes and prediabetes through PA. Firstly, PA helps to control blood glucose levels, improve insulin sensitivity, and increase insulin secretion, thereby maintaining the normal functioning of the brain and reducing the negative effects of diabetes and prediabetes on cognitive function (61, 62). Secondly, PA can release a variety of neurotransmitters (such as dopamine and endorphins), which help relieve negative emotions such as stress, anxiety, and depression, thereby improving mental health, a crucial factor for the brain’s learning and memory functions (63, 64). PA can also promote the release of brain-derived neurotrophic factors, maintain the structure and function of neurons, and improve the cognitive state of the brain (20). Finally, exercise has a certain anti-inflammatory effect, and the myogenic factor produced by skeletal muscle can delay brain aging and improve the redox state of the brain, thereby reducing the deposition of harmful substances such as *β*-amyloid and improving cognitive function (15, 65). These mechanisms interact with each other and together improve cognitive function and reduce the incidence of dementia in older adults with diabetes and prediabetes.

Building on these findings, we emphasize the importance of clinicians routinely assessing PA levels in older adults with diabetes/prediabetes and prescribing tailored, progressive exercise regimens. Secondly, we recommend that patients should actively engage in moderate to high levels of PA, increase the amount of daily activity, gradually increase the intensity of exercise, match a healthy lifestyle, have regular medical checkups to monitor blood glucose, and seek support and encouragement to work together to reduce the risk of diabetes onset and improve overall health. Our findings may offer valuable guidance for both clinicians and patients and support the integration of personalized PA plans into the routine management of diabetes to maximize the cognitive benefits of PA.

### 4.1 Strengths and limitations

For the first time, our study analyzed the association between PA and cognitive function in older people with diabetes and prediabetes by employing a sample that is nationally representative. In addition, we further explored the amount of exercise appropriate for patients with diabetes and prediabetes, grouped by PA level, as well as the dose–response correlation of PA with cognitive function. This provides a reliable basis that PA may lead to a reduced risk of cognitive decline in this population.

There are also some limitations of this study. First, self-reported questionnaires were used to assess PA and some covariates, which may introduce bias due to recall errors and information inaccuracies. The lack of device-measured PA data may attenuate the observed magnitude of the dose–response relationship. Future studies should incorporate device-based measures (e.g., accelerometers or wearable sensors) to improve accuracy and control for variability. In addition, due to the cross-sectional nature of the study, it is difficult to infer a causal relationship between PA and cognitive ability. Longitudinal cohort studies and randomized controlled trials are needed to determine the directionality and temporal sequence of these associations.

## 5 Conclusion

In conclusion, our study showed that moderate and high levels of PA can act as a key factor to maintain cognitive function in older adults with diabetes and prediabetes. Despite the need for further studies, our results strengthened support for the critical role of PA in mitigating cognitive complications associated with diabetes and prediabetes.

## Data Availability

The original contributions presented in the study are publicly available. This data can be found at: https://www.cdc.gov/nchs/nhanes/.
